# Influenza Vaccine Effectiveness Estimates among US Department of Defense Adult Beneficiaries over Four Consecutive Influenza Seasons: A Test-Negative Design Study with Different Control Groups

**DOI:** 10.3390/vaccines10010058

**Published:** 2021-12-31

**Authors:** Wenping Hu, Paul A. Sjoberg, Laurie S. DeMarcus, Anthony S. Robbins

**Affiliations:** 1 The Department of Defense Global Emerging Infections Surveillance Branch, Armed Forces Health Surveillance Division, Wright-Patterson Air Force Base, Dayton, OH 45433, USA; paul.sjoberg.1.ctr@us.af.mil (P.A.S.); laurie.demarcus.ctr@us.af.mil (L.S.D.); anthony.robbins.5@us.af.mil (A.S.R.); 2 JYG Innovations LLC, Dayton, OH 45414, USA

**Keywords:** influenza, inactivated influenza vaccine, vaccine effectiveness, test-negative design, adult

## Abstract

A test-negative design study with different control groups (influenza test-negative controls, non-influenza virus positive controls, and pan-negative controls) was conducted to assess inactivated influenza vaccine effectiveness (VE) in adults aged ≥18 years, 2016–2017 through 2019–2020 influenza seasons. A database was developed from the US Department of Defense Global Respiratory Pathogen Surveillance Program. VE was estimated using a generalized linear mixed model with logit link and binomial distribution, adjusted for confounding effects. A total of 7114 adults including 2543 medically attended, laboratory-confirmed influenza-positive cases were identified. Using influenza test-negative controls, the adjusted VE in adults was 40% [95% confidence interval (CI): 33–46%] overall, including 46% (95% CI: 36–55%) for influenza A(H1N1)pdm09, 32% (95% CI: 19–42%) for influenza A(H3N2), and 54% (95% CI: 44–62%) for influenza B. The age-stratified analysis showed that VE estimates against influenza A(H1N1)pdm09 (34%; 95% CI: −29–66%) and influenza A(H3N2) (6%; 95% CI: −60–45%) were low and non-significant for elderly adults ≥65 years of age. Overall VE estimates against any influenza or by influenza (sub)types in adults were consistent when using influenza test-negative controls, non-influenza virus positive controls, and pan-negative controls. Inactivated influenza vaccination provided moderate protection against influenza virus infection, based on the analysis from a large number of adults aged ≥18 years over multiple influenza seasons.

## 1. Introduction

Seasonal influenza primarily causes human respiratory disease in all ages. Specifically, elderly adults (≥65 years of age) are at high risk of influenza virus infection and severe influenza-related complications including hospitalization and death [1]. Influenza vaccination is considered the most effective measure available to protect against influenza viruses, combat influenza virus infection and lessen disease severity. Every year, the Department of Defense (DoD) Global Respiratory Pathogen Surveillance (DoDGRS) Program performs routine respiratory pathogen surveillance among DoD service members and their beneficiaries, allowing annual estimates of influenza vaccine effectiveness (VE) [2,3,4,5]. Annual estimates of VE are necessary as the circulating influenza viruses differ from year to year. Nevertheless, due to the small sample size, it might not be possible to accurately estimate VE in some age-stratified groups (e.g., elderly adults aged ≥65 years) in a single influenza season. There was a need thus to conduct VE analyses with greater statistical power, by combining all available data over multiple influenza seasons.

The test-negative design has been widely used in influenza VE studies since 2005 [6]. In this design, patients with influenza-like illness symptoms who tested influenza negative are commonly treated as controls. The influenza negative controls include patients who tested for other respiratory virus positive (non-influenza virus positive) and those tested for other respiratory virus negative [panel (pan)-negative]. Alternatively, the non-influenza virus positive or the pan-negative patients could be chosen as controls in a test-negative design study. In case–control studies, one of important assumptions is that control selection should be independent of exposure [7]. Nevertheless, due to potential virus interference [8], the risk of infection by a non-influenza virus could be influenced by influenza vaccination. As a result, this assumption may be violated in influenza test-negative design studies, which leads to biased control selection, consequently affecting valid VE estimates. Previous studies [6,9,10] have examined the impact of selection of different control groups on VE estimates; but the findings in their studies have been inconsistent. Feng et al. [7] conducted a meta-analysis to estimate influenza VE in the test-negative design study using alternative control groups, and found no differences in VE estimates based on the choice of control group. However, it should be noted that the conclusion drawn from the meta-analysis study [7] was based on limited 12 test-negative design studies; Moreover, there were vast differences in the age group, outpatient/inpatient settings, and countries investigated among the studies. Such variations among the studies may limit the interpretation of the meta-analysis results. Therefore, further research would undoubtedly provide more evidence to help determine right choice of control group in influenza VE studies.

Analyses reported herein are based on DoDGRS data over four consecutive influenza seasons. One objective of this study was to evaluate the effectiveness of inactivated influenza vaccine against medically attended, laboratory-confirmed influenza in adults aged ≥18 years among DoD beneficiaries in the United States seeking outpatient care over four influenza seasons (2016–2017 through 2019–2020). A secondary objective was to compare VE estimates using influenza test-negative controls to those using two other control groups (i.e., non-influenza virus positive controls and pan-negative controls) in the same study population.

## 2. Methods

### 2.1. Study Population

We used DoDGRS data over four influenza seasons from 2016–2017 to 2019–2020. All patients seeking outpatient medical care for influenza-like illness (ILI) clinical conditions were selected. ILI was defined if the patient has (1) a fever greater than or equal to 38 °C and a cough or sore throat which presents within 72 h after illness onset, or (2) physician determined ILI. Respiratory specimens were collected from ILI patients by nasopharyngeal wash or nasopharyngeal swab, and subject to testing via a multiplex respiratory pathogen panel, reverse transcription polymerase chain reaction (RT-PCR) and/or viral culture. Thus, the influenza viruses and other respiratory pathogens were identified and confirmed. Influenza virus type and subtype/lineage were identified when the respiratory specimen tested positive for influenza virus. Patients who had received at least one inactivated influenza vaccine dose 14 days or more before the onset of an ILI, were considered vaccinated. Patients who otherwise had not received vaccination before the onset of an ILI, were considered unvaccinated. Vaccination status was verified through the records from the DoD Electronic Immunization Tracking System or self-reported questionnaire for each patient. Patients with an unknown vaccination status or type, or vaccinated <14 days prior to the onset of an ILI were excluded. Vaccine type in the present study were limited to standard-dose influenza vaccine only; thus, patients who received influenza vaccine other than standard-dose influenza vaccine were excluded.

The patients in the present study were limited to adult beneficiaries from the DoDGRS program sentinel or participating sites in the United States.

### 2.2. Age Stratification

Age was classified into three groups (18–49 years, 50–64 years, and ≥65 years). The rationale for age classification was based on the fact that adults 65 years of age or older are at higher risk for influenza virus infection and severe influenza-related complications [1]. Moreover, when influenza vaccine supply is limited, vaccination efforts should focus on specific populations including all persons aged ≥50 years [11]. It was of interest to evaluate VE against influenza viruses in adults younger than 65 years of age with two further stratified age categories (18–49 years and 50–64 years). 

### 2.3. Statistical Analysis

Prior to combining data over four influenza seasons, the range of surveillance weeks in each influenza season was limited to November to April of the following year for the VE analysis, when approximately 10% or greater influenza positivity rate occurred, with an aim to minimize any potential bias due to a high ratio of influenza negative to influenza positive that would typically occur earlier or later in the influenza season.

All data was combined by influenza season, and analysis was performed using generalized linear mixed models with logit link and binomial distribution. Influenza season was treated as a random effect in the model. The odds of influenza vaccination among adults with laboratory-confirmed influenza positive (cases) were compared to the odds of influenza vaccination among adults who were tested influenza negative (controls), or to the odds of influenza vaccination among non-influenza virus positive controls or pan-negative controls. VE was calculated as (1− adjusted odds ratio) × 100%. All potential confounding factors, such as age, gender, specimen collection date, or geographical region were initially evaluated. Only those factors that changed the crude odds ratio by ≥5% were included in the generalized linear mixed models to adjust VE. In addition to overall VE estimated against any influenza viruses in the entire adult population, we estimated VE by influenza virus (sub)type in separate models [i.e., influenza A(H1N1)pdm09, influenza A(H3N2), or influenza B], and in stratified models by age category. The point estimate of VE was considered statistically significant when the lower limit of the associated 95% confidence interval (CI) did not contain zero or a negative value.

Influenza vaccination coverages were compared between two other control groups (non-influenza virus positive controls vs. pan-negative controls) using the generalized linear mixed models, adjusted for all the confounders identified in the influenza VE analysis. A *p* < 0.05 was considered statistically significant. All analyses were conducted using SAS Enterprise Guide 7.1 (SAS Institute Inc., Cary, NC, USA). 

## 3. Results

### 3.1. Patient Characteristics

The characteristics of the study population over four influenza seasons (2016–2017 to 2019–2020) is shown in Table 1. During the four influenza seasons, a total of 7114 adults were identified for the VE analysis; among whom there were 3573 (50.2%) adults aged 18–49 years, 2230 (31.4%) adults aged 50–64 years, and 1311 (18.4%) adults aged ≥65 (Table 1). Among these adults, 4071 (57.2%) were influenza vaccinated and 3043 (42.8%) were influenza unvaccinated. Vaccination coverage increased from 47.9% at age of 18–49 years to 58.6% at age of 50–64 years, reached as high as 80.3% at age of ≥65 years. Of the total 2543 influenza-positive cases, influenza A(H1N1)pdm09, influenza A(H3N2), and influenza B were 793 (31.2%), 909 (35.7%), and 590 (23.2%), respectively, with the remaining being 248 (9.8%) non-subtyped influenza A and 3 (0.1%) influenza co-infection (Table 1). Among all 4571 patients testing negative for influenza virus, 1250 (27.3%) tested positive for non-influenza virus and 3321 (72.7%) tested negative for both influenza and other respiratory viruses. The most frequently detected non-influenza viruses causing viral infection were rhinovirus/enterovirus (422; 33.8%), seasonal coronavirus (329; 26.3%), followed by human metapneumovirus (218; 17.4%).

When influenza unvaccinated, there were fewer patients (≥18 years of age) in non-influenza virus positive controls than in pan-negative controls (483 vs. 1229); likewise, fewer patients were observed (767 vs. 2092) when influenza vaccinated. Additionally, regardless of vaccination status in different age groups, there were fewer patients in non-influenza virus positive controls versus pan-negative controls (Table 2).

### 3.2. Confounding Factors Assessment

Among all potential confounders examined, month of specimen collected (i.e., one-month period from November to April of the following year), geographic region of specimen collected (i.e., the U.S. Health and Human Services Regions 1–10), and age groups (i.e., 18–49 years, 50–64 years, and ≥65 years) were the variables that changed the crude odds ratio by ≥5%. Therefore, month of specimen collected, region, and age group were included in the models to estimate overall VE and the VE stratified by influenza type/subtypes or age groups. Moreover, the same set of confounding factors were used in all models to estimate the influenza VE when using different control groups.

### 3.3. Overall VE

Using influenza test-negative controls, adjusted VE against laboratory-confirmed influenza for medically attended adults, was 40% (95% CI: 33–46%) overall, including 46% (95% CI: 36–55%) against influenza A(H1N1)pdm09, 32% (95% CI: 19–42%) against influenza A(H3N2), and 54% (95% CI: 44–62%) against influenza B (Table 3). When controls were restricted to patients who tested non-influenza virus positive the adjusted VE was 44% (95% CI: 35–52%) overall, including 50% (95% CI: 38–59%) against influenza A(H1N1)pdm09, 35% (95% CI: 19–48%) against influenza A(H3N2), and 57% (95% CI: 46–66%) against influenza B (Table 3). In addition, when controls were restricted to patients who tested pan-negative, the adjusted VE was 38% (95% CI: 30–45%) overall, including 45% (95% CI: 33–54%) against influenza A(H1N1)pdm09, 28% (95% CI: 14–40%) against influenza A(H3N2), and 52% (95% CI: 41–61%) against influenza B (Table 3).

### 3.4. VE by Age Groups

Using influenza test-negative controls, the adjusted VE estimates against any influenza were 44% (95% CI: 35–52%) in adults aged 18–49 years, and 41% (95% CI: 29–51%) in adults aged 50–64 years, then declined to 28% (95% CI: 0–49%) in adults aged ≥65 years. In comparison with those VE using influenza test-negative controls, the VE estimates against any influenza in different age groups were consistent when using pan-negative controls (Table 3). On the other hand, when using non-influenza virus positive controls, the adjusted VE against any influenza was higher (53%; 95% CI: 42–62%) in adults aged 18–49 years, compared to the VE estimates in adults aged 50–64 years (36%; 95% CI: 16–51%) or in adults aged ≥65 years (40%; 95% CI: 0–64%).

Regardless of different control groups used, the adjusted VE estimates against influenza A(H1N1)pdm09 were higher in younger adults (18–49 years and 50–64 years of age), compared to those in adults aged ≥65 years. The adjusted VE against influenza A(H3N2) was similar to that against influenza A(H1N1)pdm09 in adults aged 18–49 years; but as age increased, the VE greatly decreased. In contrast, the VE estimates against influenza B exceeded 50% in all age groups, but a wider 95% CI of the VE occurred in adults aged ≥65 years (Table 3).

### 3.5. Influenza Vaccination Coverage

There was no difference in vaccination coverage between non-influenza virus positive controls (61.4%) and pan-negative controls (63.0%) in adults (Table 4). By age groups, vaccination coverage was higher (55.0 vs. 51.7%; *p* = 0.012) in adults aged 18–49 years for non-influenza virus positive controls vs. pan-negative controls; however, no statistically different vaccine coverages were found in adults aged 50–64 years (61.1 vs. 66.0%) and in adults aged ≥65 years (61.4 vs. 63.0%) between these two control groups. 

## 4. Discussion

We report the pooled estimates of VE against medically attended, laboratory-confirmed influenza in adults over the 2016–2020 influenza seasons (Table 3). As analyzed using influenza test-negative controls, significant protection of standard-dose influenza vaccine against any influenza was found in adults (40%), while by influenza (sub)type, the protection was higher against influenza A(H1N1)pdm09 and influenza B, but lower against influenza A(H3N2). Moreover, the age-stratified analysis indicated the overall VE against any influenza viruses was lower in adults aged ≥65 years compared with younger adults (18–49 years or 50–64 years of age).

Previously, we conducted the VE analysis in children aged 6 months-17 years that included DoDGRS data in outpatient settings within the same seasons [12]. It was shown that the VE against influenza in children was 42% (95% CI: 37–47%) overall, including 55% (95% CI: 47–61%) against influenza A(H1N1)pdm09, 37% (95% CI: 28–45%) against influenza A(H3N2), and 49% (95% CI: 41–55%) against influenza B. In comparison, influenza vaccine offered similar protections in adults overall and against influenza B, and lower but comparable protections against influenza A(H1N1)pdm09 and influenza A(H3N2).

By age groups, Belongia et al. [13] found in their meta-analysis of test-negative design studies from 2004–2015, that the pooled VE against medically attended influenza in working age adults (20–64 years old) and older adults (>60 years) were 73% (95% CI: 52–84%) and 62% (95% CI: 36–78%) for influenza A(H1N1)pdm09, 35% (95% CI: 14–51%) and 24% (95% CI: −6–45%) for influenza A(H3N2), and 54% (95% CI: 16–75%) and 63% (95% CI: 33–79%) for influenza B, respectively. More recently, Russell et al. [14] combined data among outpatients aged ≥18 years from the US Flu VE Network over five influenza seasons (2011–2012 through 2015–2016). Their analysis showed that the adjusted VE in adults aged 18–49 years and ≥65 years were 48% (95% CI: 38–57%) and 49% (95% CI: 22–66%) for influenza A(H1N1)pdm09, 21% (95% CI: 10–32%) and 14% (95% CI: −14–36%) for influenza A(H3N2), and 55% (95% CI: 45–63%) and 62% (95% CI: 44–74%) for influenza B, respectively. Compared to the findings from these studies [13,14], we found similar VE estimates against influenza B in adults with different ages. Moreover, lower VE estimate against influenza A(H3N2) was consistently observed in adults aged ≥65 years than in adults aged 18–49 years. However, in the present study, the VE estimate against influenza A(H1N1)pdm09 in adults aged ≥65 years was lower and non-significant. Recent studies in the US [15,16,17], which performed VE analysis each season individually over the three seasons from 2016–2017 to 2018–2019, have also shown low and non-significant effectiveness of influenza vaccination against influenza A(N1H1)pdm09 and influenza A(H3N2) in adults aged ≥65 years, with the exception of no assessment of VE against influenza A(H1N1)pdm09 being reported during 2016–2017 influenza season [15].

Our findings clearly demonstrated inactivated influenza vaccine was less effective against influenza A(H1N1)pdm09 and influenza A(H3N2) in adults aged ≥65 years. Age is one of the major host factors (e.g., age, history of infection and prior vaccination, health status, etc.) to determine influenza vaccine response [18]. The decreased VE in adults aged ≥65 years is usually attributed to immunosenescence, a gradual decline in immune function as age advances [19,20]. Indeed, the decline of immune function in older people would lead to greater susceptibility to influenza virus and affect their ability to respond to influenza vaccination. The protection provided by influenza vaccine is based on induction of antibodies. Goodwin et al. [21] showed that antibody response to influenza vaccine in the elderly is considerably lower than in younger adults. In addition, there is increasing evidence suggesting that intra-season waning of influenza vaccine protection occurs [22,23], and such waning immunity is more pronounced for older adults [24]. 

Several studies have suggested that repeated vaccination would impact vaccine protection against influenza virus, which might consequently alter VE estimates [25,26]. The underlying immunologic mechanisms for potential vaccine interference are not well understood [27]. Khurana et al. [28] observed a significant negative impact of repeat vaccination on antibody affinity maturation, which may contribute to lower VE of influenza vaccine. Further, age may confound the effect of prior vaccination history on the response to influenza vaccination [29]. Annual influenza vaccination is recommended for specific populations at high risk, including older adults [11]. Older people may thus have more opportunities to receive repeated influenza vaccination. The highest influenza vaccination coverage was evident in adults aged ≥65 years (80.3%) in the present study, which is consistent with the previous findings in the US study [30]. Therefore, greater potential impact of repeated vaccination on VE might be expected in adults aged ≥65 years, compared to younger adults.

The impact of three control groups on VE estimates in adults has been evaluated in the present study. Overall VE or VE by influenza (sub)types estimated using non-influenza virus positive controls or pan-negative controls were similar, or comparable to those estimated using test-negative controls. Additionally, the 95% CI of VE estimates widely overlapped (Table 3). Nevertheless, VE estimates by age groups presented some variations based on the three control groups used. We found that the influenza test-negative and the pan-negative control groups gave more consistent VE estimates (Table 3). It appeared that VE estimates using non-influenza virus positive controls were higher than VE estimates using influenza test-negative controls or pan-negative controls in adults aged 18–49 years and in all adults, but lower in adults aged 50–64 years. van Doorn et al. [10] found in their study conducted in the Dutch population from 2003 to 2014, that influenza estimates in both the main and subgroup analyses using non-influenza virus positive controls were the highest among three different control groups used. Additionally, highest VE estimate using non-influenza virus positive controls was observed in an earlier study [31]. In contrast, Feng et al. [6] estimated the VE over three influenza seasons (2010–2013) in outpatient settings, and observed that VE estimates using each of the three control groups were consistent overall or when stratified by age groups. In the study of Pierse et al. [9], similar VE estimates were derived using influenza test-negative controls and non-influenza virus positive controls.

Considering the variations of the influenza VE estimates observed by age group using non-influenza virus positive controls vs. influenza test-negative controls or pan-negative controls, it was observed that when stratified by age groups, particularly for the adults aged ≥65 years, there was much smaller proportion of patients in non-influenza virus positive controls (Table 2). The smaller sample size might explain the variations of VE estimated using non-influenza virus positive controls compared to those using influenza test-negative controls or pan-negative controls. Influenza viruses co-circulate with other respiratory viruses in influenza season. When influenza vaccinated, the risk of a non-influenza respiratory virus infection may increase due to virus interference via a biological mechanism of temporary nonspecific immunity, resulting in a higher proportion of vaccinated patients in non-influenza virus positive controls and an overestimation of VE [6,10]. In the present study, we observed no difference in the vaccination coverage between the non-influenza virus positive controls and pan-negative controls in adults. However, when examined by age group, higher vaccination coverage (*p* = 0.012) in adults aged 18–49 years was found in non-influenza virus positive controls vs. pan-negative controls, but this has not been seen in adults aged 50–64 years and in adults aged ≥65 years (Table 4). Furthermore, it appeared that the differences in influenza vaccination coverage did not consistently reflect the changes of VE estimates in different age groups. Therefore, it is unlikely that influenza vaccination would be associated with the detection of non-influenza respiratory viruses. Previous studies have not shown the association between influenza vaccination and the detection of non-influenza respiratory virus [6,32]. Nevertheless, we could not rule out the possibility that influenza vaccination may affect the susceptibility to a certain or several non-influenza respiratory viruses. There is increasing evidence suggesting that there are potential virus interferences or virus interactions, which impact the dynamics of seasonal influenza [8,33].

This study is subject to several limitations. First, our efforts to estimate the effect of vaccination rely on the DoD surveillance platform for data acquisition. When ascertaining vaccine status based on the self-reported questionnaire, potential non-differential misclassification could occur. In addition, the specimens collected in DoDGRS program were through routine outpatient clinical care. Before evaluating the validity of using such routinely collected data in VE analysis, the possibility of potential selection bias could not be ruled out. Second, as discussed above, there is potential impact of repeat vaccination on the VE estimates. However, the models used for VE analysis in the present study did not account for prior exposure to influenza virus antigens by repeated influenza vaccination or natural influenza virus infection in previous seasons. Similarly, there were other unmeasured confounding factors such as health status of patients. Specifically for the older adults, the age-associated changes in the immune response to influenza vaccination is influenced by the increasing level of frailty [34]. Therefore, such unmeasured factors could not be ruled out as a possible alternative explanation for the findings. Third, there were an insufficient number of patients who received high-dose influenza vaccination, thereby those patients were excluded for analysis in the present study. We were unable to compare effectiveness of high-dose and standard-dose influenza vaccine against medically attended, laboratory-confirmed influenza in adults aged ≥65 years. Ng et al. [35] found that high-dose influenza vaccine had greater antibody responses than the standard-dose influenza vaccine. In addition, studies comparing high-dose to standard-dose influenza vaccine have demonstrated increased effectiveness via high-dose influenza vaccination [36,37]. Given the limitation in the effectiveness of standard-dose influenza vaccine among the elderly, it is critical to consider alternative influenza vaccine strategies including high-dose influenza vaccine for this population [38].

## 5. Conclusions

In conclusion, the present study demonstrated moderate effectiveness of inactivated influenza vaccination in adults aged ≥18 years against medically attended, laboratory-confirmed influenza virus infection. We found the VE against influenza A(H3N2) was lower in adults aged 50–64 years than in adults aged 18–49 years. Moreover, we observed low and non-significant VE against influenza A(H1N1)pdm09 and influenza A(H3N2) in adults aged ≥65 years. Using three different control groups, overall VE estimates against any influenza or by influenza (sub)types were consistent in adults. However, when stratified by age group, some variations of VE estimates occurred between influenza test-negative controls (or pan-negative controls) and non-influenza virus positive controls, probably due to limited number of patients included in non-influenza virus positive controls. Further research is needed to clarify the observed differences in number of patients included in different control groups. Our findings support the use of test-negative design (case vs. test-negative control) to evaluate the effectiveness of influenza vaccination in outpatient settings.

## Figures and Tables

**Table 1:** Characteristics of study population used for vaccine effectiveness analysis over four influenza seasons (2016–2017 to 2019–2020).

	Overall	2016–2017	2017–2018	2018–2019	2019–2020
Characteristic	N (%)	N (%)	N (%)	N (%)	N (%)
Gender					
Male	2360 (33.17)	225 (28.96)	551 (32.58)	865 (36.12)	719 (31.94)
Female	4754 (66.83)	552 (71.04)	1140 (67.42)	1530 (63.88)	1532 (68.06)
Age					
18–49 years	3573 (50.22)	471 (60.62)	828 (48.97)	1058 (44.18)	1216 (54.02)
50–64 years	2230 (31.35)	249 (32.05)	566 (33.47)	706 (29.48)	709 (31.5)
≥65 years	1311 (18.43)	57 (7.34)	297 (17.56)	631 (26.35)	326 (14.48)
Month of illness					
November	231 (3.25)	14 (1.8)	53 (3.13)	0 (0)	164 (7.29)
December	975 (13.71)	120 (15.44)	246 (14.55)	272 (11.36)	337 (14.97)
January	1925 (27.06)	170 (21.88)	544 (32.17)	587 (24.51)	624 (27.72)
February	2009 (28.24)	220 (28.31)	484 (28.62)	721 (30.1)	584 (25.94)
March	1575 (22.14)	174 (22.39)	247 (14.61)	612 (25.55)	542 (24.08)
April	399 (5.61)	79 (10.17)	117 (6.92)	203 (8.48)	0 (0)
Geographic region ^a^					
Region 1	23 (0.32)	2 (0.26)	2 (0.12)	2 (0.08)	17 (0.76)
Region 2	479 (6.73)	90 (11.58)	182 (10.76)	115 (4.8)	92 (4.09)
Region 3	489 (6.87)	87 (11.2)	177 (10.47)	59 (2.46)	166 (7.37)
Region 4	931 (13.09)	129 (16.6)	302 (17.86)	236 (9.85)	264 (11.73)
Region 5	996 (14)	18 (2.32)	363 (21.47)	264 (11.02)	351 (15.59)
Region 6	2554 (35.9)	167 (21.49)	305 (18.04)	1389 (58)	693 (30.79)
Region 7	246 (3.46)	54 (6.95)	57 (3.37)	29 (1.21)	106 (4.71)
Region 8	682 (9.59)	90 (11.58)	172 (10.17)	157 (6.56)	263 (11.68)
Region 9	471 (6.62)	76 (9.78)	97 (5.74)	66 (2.76)	232 (10.31)
Region 10	243 (3.42)	64 (8.24)	34 (2.01)	78 (3.26)	67 (2.98)
Vaccine status					
Vaccinated	4071 (57.23)	330 (42.47)	841 (49.73)	1596 (66.64)	1304 (57.93)
Unvaccinated	3043 (42.77)	447 (57.53)	850 (50.27)	799 (33.36)	947 (42.07)
Influenza					
A(H1N1)pdm09	793 (11.15)	4 (0.51)	99 (5.85)	185 (7.72)	505 (22.43)
A(H3N2)	909 (12.78)	264 (33.98)	419 (24.78)	208 (8.68)	18 (0.8)
A/not subtyped	248 (3.49)	0 (0)	0 (0)	242 (10.1)	6 (0.27)
B	590 (8.29)	78 (10.04)	257 (15.2)	18 (0.75)	237 (10.53)
Dual influenza	3 (0.04)	0 (0)	3 (0.18)	0 (0)	0 (0)
Non-influenza	4571 (64.25)	431 (55.47)	913 (53.99)	1742 (72.73)	1485 (65.97)

^a^ The U.S. Health and Human Services Regions 1–10, except for Guam, Alaska, and Hawaii.

**Table 2:** Characteristics of patients by vaccination status in the test-negative design study with different control groups.

	Unvaccinated							Vaccinated						
		Control Groups							Control Groups					
		Influenza		Non-Influenza					Influenza		Non-Influenza			
		Test-Negative		Virus Positive		Pan-Negative			Test-Negative		Virus Positive		Pan-Negative	
	Case	Control	Case (%)	Control	Case (%)	Control	Case (%)	Case	Control	Case (%)	Control	Case (%)	Control	Case (%)
18–49 years														
Influenza A(H1N1)pdm09	227	1068	17.53	297	43.32	771	22.75	184	1189	13.40	363	33.64	826	18.22
Influenza A(H3N2)	292	1068	21.47	297	49.58	771	27.47	169	1189	12.44	363	31.77	826	16.98
Influenza B	217	1068	16.89	297	42.22	771	21.96	111	1189	8.54	363	23.42	826	11.85
Any influenza ^a^	794	1068	42.64	297	72.78	771	50.73	522	1189	30.51	363	58.98	826	38.72
50–64 years														
Influenza A(H1N1)pdm09	160	473	25.28	148	51.95	325	32.99	153	863	15.06	232	39.74	631	19.52
Influenza A(H3N2)	148	473	23.83	148	50.00	325	31.29	158	863	15.48	232	40.51	631	20.03
Influenza B	122	473	20.50	148	45.19	325	27.29	85	863	8.97	232	26.81	631	11.87
Any influenza ^a^	450	473	48.75	148	75.25	325	58.06	444	863	33.97	232	65.68	631	41.30
≥65 years														
Influenza A(H1N1)pdm09	19	171	10.00	38	33.33	133	12.50	50	807	5.83	172	22.52	635	7.30
Influenza A(H3N2)	35	171	16.99	38	47.95	133	20.83	107	807	11.71	172	38.35	635	14.42
Influenza B	21	171	10.94	38	35.59	133	13.64	34	807	4.04	172	16.50	635	5.08
Any influenza ^a^	87	171	33.72	38	69.60	133	39.55	246	807	23.36	172	58.85	635	27.92
≥18 years														
Influenza A(H1N1)pdm09	406	1712	19.17	483	45.67	1229	24.83	387	2859	11.92	767	33.54	2092	15.61
Influenza A(H3N2)	475	1712	21.72	483	49.58	1229	27.88	434	2859	13.18	767	36.14	2092	17.18
Influenza B	360	1712	17.37	483	42.70	1229	22.66	230	2859	7.45	767	23.07	2092	9.91
Any influenza ^a^	1331	1712	43.74	483	73.37	1229	51.99	1212	2859	29.77	767	61.24	2092	36.68

^a^ Including influenza A(H1N1)pdm09, influenza A(H3N2), influenza B, influenza A/not subtyped, and influenza co-infection.

**Table 3:** Adjusted vaccine effectiveness in adults aged ≥18 years.

	Controls					
	Influenza Test-Negative		Non-Influenza Virus Positive		Pan-Negative	
	VE (%) ^a^	95% CI (%)	VE (%) ^a^	95% CI (%)	VE (%) ^a^	95% CI (%)
18–49 years						
Influenza A(H1N1)pdm09	41	25–53	52	36–64	35	16–49
Influenza A(H3N2)	42	26–54	51	34–64	36	18–50
Influenza B	55	41–65	62	48–72	51	35–63
Any influenza ^b^	44	35–52	53	42–62	39	28–48
50–64 years						
Influenza A(H1N1)pdm09	53	38–65	47	24–62	57	41–68
Influenza A(H3N2)	26	1–45	13	−30–41	26	−1–46
Influenza B	56	38–68	52	29–68	57	39–70
Any influenza ^b^	41	29–51	36	16–51	43	30–53
≥65 years						
Influenza A(H1N1)pdm09	34	−29–66	41	−26–73	27	−46–63
Influenza A(H3N2)	6	−60–45	15	−82–60	−4	−84–41
Influenza B	60	19–80	65	18–85	55	6–78
Any influenza ^b^	28	0–49	40	0–64	24	−8–47
≥18 years						
Influenza A(H1N1)pdm09	46	36–55	50	38–59	45	33–54
Influenza A(H3N2)	32	19–42	35	19–48	28	14–40
Influenza B	54	44–62	57	46–66	52	41–61
Any influenza ^b^	40	33–46	44	35–52	38	30–45

^a^ VE: vaccine effectiveness, adjusted for months of specimen collecting date, region of specimen collected, and age groups. ^b^ Including influenza A(H1N1)pdm09, influenza A(H3N2), influenza B, influenza A/not subtyped, and influenza co-infection.

**Table 4:** Influenza vaccine coverage among patients tested non-influenza virus positive and patients tested pan-negative.

	Non-Influenza Virus Positive Controls		Pan-Negative Controls		*p*-Value
	Total	Vaccinated (%)	Total	Vaccinated (%)	
18–49 years	660	363 (55.0)	1597	826 (51.7)	0.012
50–64 years	380	232 (61.1)	956	631 (66.0)	0.198
≥65 years	210	172 (81.9)	768	635 (82.7)	0.709
≥18 years	1250	767 (61.4)	3321	2092 (63.0)	0.245

## Data Availability

All data is presented in this article. For further information contact the corresponding author.
